# Potential Effects on Mental Health Status Associated with Occupational Exposure to Pesticides among Thai Farmers

**DOI:** 10.3390/ijerph19159654

**Published:** 2022-08-05

**Authors:** Parichat Ong-Artborirak, Waraporn Boonchieng, Yuwayong Juntarawijit, Chudchawal Juntarawijit

**Affiliations:** 1 Faculty of Public Health, Chiang Mai University, Chiang Mai 50200, Thailand; 2 Center of Excellence in Community Health Informatics, Chiang Mai University, Chiang Mai 50200, Thailand; 3 Faculty of Nursing, Naresuan University, Phitsanulok 65000, Thailand; 4 Faculty of Agriculture Natural Resources and Environment, Naresuan University, Phitsanulok 65000, Thailand

**Keywords:** pesticide, farmer, symptom, mental health, psychiatric disorder

## Abstract

Pesticide-related mental health issues in Thailand, an upper-middle-income country, are not well known. This study aimed to investigate the association between the history of occupational exposure to pesticides and the mental health of Thai farmers. A cross-sectional study was carried out in the areas around Chiang Mai, a large city in Northern Thailand, between June 2020 and January 2021. A total of 6974 farmers from six districts were interviewed to determine whether they regularly experienced symptoms related to mental health by the Self-Reporting Questionnaire (SRQ-20) as well as their lifetime history of agricultural pesticide exposure from 31 active ingredients and five functional categories: insecticides, herbicides, fungicides, rodenticides, and molluscicides. The cut-off of 6 was used to evaluate probable mental disorder. Most of the farmers under investigation were men (53.8%), with a mean age of 55.2 (11.7) years, and were involved mainly in the planting of rice, fruit, and vegetables. About 86.7% reported having used pesticides on their crops at some point in their lives—mostly glyphosate, paraquat, 2,4-D, methomyl, and carbofuran. All functional groups, as well as pesticide classes like organochlorines, organophosphates, and carbamates, were significantly associated with a higher risk of probable mental disorder based on exposure duration, frequency, personal protective equipment usage, and hygienic behavior. In a model with multiple pesticides, there was an association between mental disorder and exposure to endosulfan (AOR = 2.27, 95%CI = 1.26–4.08) and methyl parathion (AOR = 2.26, 95%CI = 1.26–4.06). Having previously reported pesticide poisoning symptoms was related to mental disorder (AOR = 7.97, 95%CI = 5.16–12.31), the findings provided evidence of pesticide exposure posing a risk to farmers’ mental health, particularly long-term and high-intensity exposure.

## 1. Introduction

Globally, the issue of mental health is a major public health concern. Mental disorders, most commonly depression and anxiety, affect an estimated 970 million people worldwide [1]. Suicide is known to result from neuropsychiatric disorders such as depression. According to data from the 4th Thai national mental health survey in 2013, the lifetime prevalence of any mental disorder was 7.4%, with the highest reported in the north (9.3%) [2]. Correspondingly, the suicide rate per 100,000 Thai people in each year from 2017–2020 was reported to be 6.0, 6.3, 6.6, and 7.4, respectively, whereas in Chiang Mai, a province in the Northern Thailand, the rates were found to be 11.2, 10.2, 11.2, and 14.6, respectively [3]. This pattern indicated an increasing burden of mental disorders in Thailand. Socio-demographic characteristics and environmental conditions are well known to have an impact on mental health.

Currently, there is mounting evidence of a link between mental disorders and occupational pesticide exposure. A previous study revealed that farmers had significantly higher rates of anxiety/insomnia and severe depression than the controls [4]. Farmers are regularly directly exposed to pesticides via mainly inhalation and skin contact, which is a major cause of pesticide poisonings, particularly organophosphates and carbamates. Chronic pesticide exposure can inhibit acetylcholinesterase activity and generate neuropsychiatric disorders as well as adverse health effects [5,6,7]. Several studies have revealed a link between pesticide poisoning and mental disorders, particularly depression and anxiety [8,9,10,11]. Furthermore, numerous studies have discovered an elevated risk of depression and anxiety associated with exposure to pesticides, such as organochlorines, organophosphates, carbamates, pyrethroids, and herbicides like phenoxy and paraquat dichloride [9,10,11,12,13,14,15,16].

However, other pesticides such as neonicotinoid and avermectin insecticides, fungicides, rodenticides, molluscicides, have received little empirical attention. Data on mental health related to occupational pesticide exposure in Thailand, where multiple pesticides are widely used, is scarce. As a result, the goals of this study were to examine the relationship between lifetime exposure history to pesticides and mental health among Thai farmers. The expected outcome of the study can be used in policy planning and advocacy in order to resolve mental health issues and improve mental well-being and quality of life.

## 2. Materials and Methods

### 2.1. Study Design and Subjects

This cross-sectional study was conducted in Chiang Mai province, a large economic center and agricultural community in Northern Thailand, between June 2020 and January 2021 as part of the Agricultural Health of Thai Farmers project. Cluster sampling was used to select six districts out of a total of 25 in the Chiang Mai province for the recruitment of all farmers in agricultural areas. There are approximately 180,000 farms in the province, 32,000 of which are located in the six districts we investigated at for our study, and the majority of them are rice and fruit farms [17]. Public health officers and health volunteers in the areas under their responsibility were assigned to select the main farmers, excluding those in animal agriculture, in each household. The inclusion criteria were as follows: (1) male or female over the age of 20, (2) work experience of at least one year, (3) ability to read and speak the local language, and (4) willingness to participate in the study. There were 7277 farmers who participated in the study; however, some of them were excluded from the analysis due to a lack of written informed consent, serious medical issues, and incomplete questionnaire data. Finally, this study examined data from 6974 farmers. The Naresuan University Institutional Review Board approved the study protocol (COA No. 657/2019).

### 2.2. Data Collection

Face-to-face interviews were conducted by public health officers and health volunteers who had been trained in interviewing and administering an online questionnaire. Prior to use, the entire questionnaire was tested. The study provided an interviewer identification number that can be used to inspect and recheck the received data for quality control. The questionnaire contained the following sections: (a) socio-demographics (sex, age, marital status, education, income, smoking, drinking alcohol, proximity to a farm); (b) work characteristics (current job duration, type of crops, number of agriculture areas, frequency of entering farm, history of any pesticide use for their crops); and (c) other history about pesticides (only pesticide users), including self-reported symptoms of any pesticide poisonings after 24-h exposure, and practice in pesticide use. The practice included use of personal protective equipment (PPE) (chemical mask, gloves, long-sleeve shirt and long pants, and boots), and taking a bath and changing clothes immediately after pesticide use, which were categorized as always, sometimes, and rarely/never.

Questions about history of any pesticide use was modified from the Agricultural Health Study [13,18], as well as pesticides were selected based on their high toxicity and high quantity of import into Thailand [19]. We collected information on lifetime exposure of five functional pesticide groups (herbicides, insecticides, fungicides, rodenticides, and molluscicides) and 31 active ingredients, with exposure duration (≤1, 2–5, 6–10, 11–20, and >20 years) and exposure frequency (<5, 5–9, 10–19, 20–39, 40–59, 60–150, and >150 days per year). Previous research on a sample of participants in the Agricultural Health Study showed good reliability about ever-/never-use of specific pesticides with duration and frequency of pesticide use [20]. To confirm the type of pesticides used, example trade names and pictures of each pesticide were used, along with questions about exposure history.

To estimate cumulative lifetime pesticide exposure, we adapted a semi-quantitative exposure algorithm developed for low-middle-income contexts from previous studies [21,22], combining lifetime application days, PPE use, and hygienic behaviors (Equation (1)).

Cumulative lifetime exposure = Lifetime application days × PPE score × Bathing score(1)

The lifetime application days were computed by multiplying the average number of days per year (the midpoints of the category) by the total number of years of pesticide use (the midpoints of the category) [14]. The PPE score was calculated to adjust the exposure intensity (Equation (2)).

PPE score = 0.1PPE_mask_ + 0.1PPE_goggles_ + 0.4PPE_hand_ + 0.3PPE_shirt&pants_ + 0.1PPE_Boots_ (2)

The farmer’s reported use of PPE was given a score based on the following categories: always (mask and boots = 0.1, hand = 0.2, and shirt & pants = 0.3), sometimes (mask and boots = 0.55, hand = 0.6, and shirt & pants = 0.65), and rarely/never (mask, goggles, hand, shirt & pants, and boots = 1) [21]. We assumed that Thai farmers rarely wear goggles. An example of PPE score estimation, a pesticide applicator reported that he used gloves sometimes, long-sleeve shirt & pants always, and rubber boots always; he never used a mask with carbon filter. As a result, the applicator would have a PPE score of 0.54, adjusting or decreasing about half of the exposure. Bathing and changing clothes immediately after pesticide use was scored as follows: always = 0.8, sometimes = 0.9, and rarely/never = 1 [21].

The cumulative days for any pesticide use were computed by summing the lifetime application days of each of the five functional groups. Lifetime application days for pesticide classes were also created by combining data for active ingredients, including 5 herbicides, 21 insecticides, and 5 fungicides. This resulted in four classes of herbicides (glycine, bipyridylium, phenoxy, and chloroacetamide/anilide), six classes of insecticides (organochlorine, organophosphate, carbamate, pyrethroid, neonicotinoid, and avermectin), and three classes of fungicides (phenylamide, inorganic, and dithiocarbamate). Cumulative exposure to any pesticide, herbicides, insecticides, fungicides, glycines, and bipyridyliums was categorized in three groups: low exposure (the median), medium exposure (the median to the 90th percentile), and high exposure (>90th percentile). The split of cumulative pesticide exposure into three levels presumably reduces the risk of misclassification [14]. However, due to low reported pesticide use, lifetime exposure to rodenticides, molluscicides, phenoxies, chloroacetamides/anilide, organochlorines, organophosphates, carbamates, avermectins, and dithiocarbamates was collapsed into two groups by the median. Whereas the exposure to pyrethroids, neonicotinoids, phenylamides, and inorganic fungicides was not divided.

While screening for mental health issues related to pesticide exposure, all participants were assessed by the Self Reporting Questionnaire (SRQ-20) recommended by the World Health Organization (WHO), which is suitable as an interviewer-administered questionnaire for evaluating psychiatric disorders [23]. The questionnaire contained 20 dichotomous items (yes = 1/no = 0) about neurotic symptoms like depression and anxiety, yielding a score range of 0–20. All famers were asked about the symptoms they regularly experienced in the month preceding the interview in order to confirm whether they exist on a regular basis and to reduce potential misclassification. A probable mental disorder was defined as a score of ≥6 during the survey, which is the optimal cutoff score for middle-income countries [24,25]. Because of the low prevalence of farmers disclosing any regularly neurotic symptoms, the 90th and 95th percentiles (score > 1 and >2) was used as another outcome in this study to analyze the association with pesticide exposure.

### 2.3. Data Analysis

Initially, the imported data was checked and cleaned. The SPSS Version 17 software (SPSS Inc., Chicago, IL, USA), licensed from Chiang Mai University, was used for statistical analysis. An association between pesticide exposure and mental health outcomes (symptoms ≥ 2, ≥3, and ≥6 scores) were determined using binary logistic regression analysis. Our study found that demographic characteristics—sex (male, female), age (≤ 40, 41–50, 51–60, >60 years), marital status (married, single/divorced), education (no, primary, secondary or higher), smoking (no, yes), and drinking alcohol (no, yes)—were associated with mental health outcomes among farmers in univariable analysis. We selected these covariates to test the associations for cumulative exposure to any pesticides, functional groups, and chemical classes. Some variables (income, farm proximity, and farm size) which did not have a significant presence in the analysis were not adjusted. In each analysis, farmers not exposed to any pesticide were used as a reference group. In addition, a history of pesticide poisonings was examined in relation to mental health outcomes among participants who used pesticides. To test the associations for pesticide active ingredients, a history of pesticide use in agriculture (no/yes) was added to adjust the model. However, to increase the statistical power, only individual active ingredients reported as being utilized by at least 500 farmers were analyzed. Consequently, out of a total of 31 compounds, we assessed 11 of them (glyphosate, paraquat, 2,4-D, endosulfan, chlorpyrifos, methyl parathion, methomyl, carbofuran, carbaryl, abamectin/emamectin benzoate, mancozeb). We looked at both a single model (in which each active ingredient was tested separately) and a multiple model (in which multiple active ingredients were tested all at once). The highest value for the variance inflation factor (VIF) in each regression model was found to be 1.6. A *p*-value of 0.05 was considered statistically significant, and the odds ratio (OR) with a 95% confidence interval (CI) was reported. In addition, the correlation between lifetime application days of functional groups, chemical classes, and individual active ingredients was investigated using Spearman’s rank correlation coefficient (r_s_).

## 3. Results

The socio-demographic and work characteristics of the farmers are shown in Table 1. In the study area, the most popular plant was rice, followed by fruits (mainly longan, banana, and mango), vegetables (mainly shallot, garlic, cabbage, and baby corn), and flowers (mainly orchid, rose, jasmine, and marigold). Most farmers (86.7%) had the history of pesticide application, mainly sprayer (75.1%) and mixer (44.9%). Of the pesticide users, 17.3% reported at least one symptom of pesticide poisoning 24-h after application. The common symptoms of pesticide poisonings experienced by the participants were headache, dizziness, excessive sweating, fatigue/tiredness, and skin irritation.

The history of pesticide use reported by farmers is presented in Table 2. Regarding duration and frequency of pesticide exposure, the majority of farmers reported the period of time of the use of individual pesticides as follows: (a) more than 20 years and 20–39 days/year with monocrotophos; (b) more than 20 years and less than 5 days/year with alachlor, endosulfan, and permethrin/cypermethrin; (c) 11–20 years and less than 5 days/year with profenofos, and imidacloprid; (d) 6–10 years and 60–150 days/year with dieldrin/aldrin; (e) 6–10 years and 10–19 days/year with chlorpyrifos, abamectin/emamectin benzoate; (f) 6–10 years and less than 5 days/year with glyphosate, paraquat, 2,4-D, butachlor/propanil, DDT, methyl parathion, dichlorvos, carbaryl, metalaxyl, bordeaux mixture/copper sulfate (CuSO_4_), maneb/zineb, and propineb; (g) 2–5 years and 10–19 days/year with heptachlor, methamidophos, and carbosulfan; (h) 2–5 years and 5–9 days/year with mevinphos, and methomyl; and (i) 2–5 years and less than 5 days/year with chlordane, EPN, dicrotophos, carbofuran, and mancozeb.

The prevalence of neurotic symptoms and mental disorder among farmers assessed by the Self-Reporting Questionnaire (SRQ-20) is shown in Table 3. The highest prevalence of neurotic symptoms was sleeping problems (8.0%), headache (6.1%), and lack of appetite (4.2%). Correlation coefficients greater than 0.3 were found between the following pesticides: insecticides and herbicides (r_s_ = 0.70), insecticides and fungicides (r_s_ = 0.69), glyphosate and paraquat (r_s_ = 0.66), fungicides and avermectin (r_s_ = 0.55), molluscicides and endosulfan (r_s_ = 0.51), herbicides and fungicides (r_s_ = 0.49), molluscicides and organochlorines (r_s_ = 0.44), rodenticides and molluscicides (r_s_ = 0.39), endosulfan and methomyl (r_s_ = 0.33), and molluscicides and methomyl (r_s_ = 0.31), respectively.

Association of pesticide exposure with mental health symptoms and mental disorder after being adjusted for sex, age, marital status, education, smoking, and alcohol consumption is presented in Table 4. Among farmers who had used pesticides, having previously reported pesticide poisoning symptoms was a risk factor for probable mental disorder (AOR = 7.97, 95%CI = 5.16–12.31). Overall pesticide exposure and each functional group—herbicides, insecticides, fungicides, rodenticides, and molluscicides—was significantly associated with a higher risk of probable psychiatric disorder depending on duration of exposure, frequency of exposure, use of PPE, and hygienic behavior. Farmers who were highly exposed to each pesticide class (cumulative exposure above median)—glycines (glyphosate), bipyridyliums (paraquat), phenoxies (2,4-D), chloroacetamides/anilides, organophosphates, carbamates, dithiocarbamates, and inorganic fungicide (bordeaux mixture/CuSO_4_)—had a higher risk of mental disorder than farmers who did not use pesticide (*p* < 0.05). It was also found for organochlorines both low and high exposure (*p* < 0.05). Whereas a mental disorder was marginally associated with exposure to pyrethroids (permethrin/cypermethrin) (*p* = 0.082), neonicotinoid (imidacloprid) (*p* = 0.076), avermectin (abamectin/emamectin benzoate) (*p* = 0.058), and phenylamide (metalaxyl) (*p* = 0.059).

**Figure 1:**
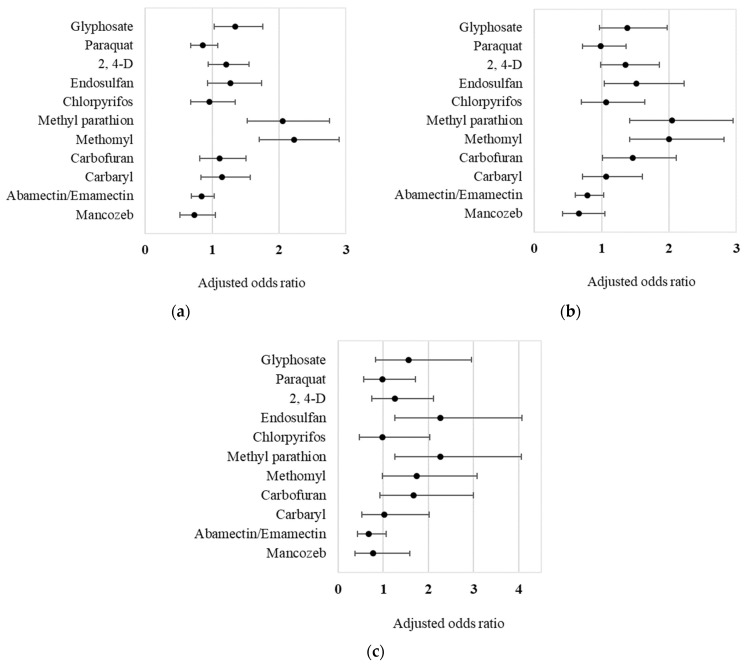
Testing multiple pesticide active ingredients with neurotic symptoms among farmers: (**a**) At P90 (score ≥ 2); (**b**) At P95 (score ≥ 3); (**c**) At score ≥ 6 (probable mental disorder), adjusted for history of pesticide use in agriculture, sex, age, marital status, education, smoking, and drinking alcohol.

Association of specific pesticide exposure with neurotic symptoms and mental disorder after controlling for history of pesticide use in agriculture, sex, age, marital status, education, smoking, and alcohol consumption is shown in Table 5. In single a pesticide model, a history of pesticide exposure to eight active ingredients—glyphosate, paraquat, 2,4-D, endosulfan, methyl parathion, methomyl, carbofuran, and carbaryl—was found to be a significant factor of probable mental disorder (*p* < 0.05), with methyl parathion having the highest odds ratio (AOR = 4.44, 95%CI = 2.71–7.29). In a multi-pesticide model in which many active ingredients were examined simultaneously, there was a strong association between mental disorder and exposure to endosulfan (*p* < 0.01) and methyl parathion (*p* < 0.01), with a marginal association for methomyl (*p* = 0.057) and carbofuran (*p* = 0.088). In addition, exposure to glyphosate, methyl parathion, and methomyl were significantly associated with having at least two neurotic symptoms (*p* < 0.05). Exposure to endosulfan, methyl parathion, methomyl, and carbofuran were significantly associated with having at least three neurotic symptoms (*p* < 0.05). Forest plots of the odds ratios for the multi-pesticide model are illustrated in Figure 1.

## 4. Discussion

### 4.1. Principal Findings

After controlling for socio-demographic factors, our findings highlighted the possibility of mental health problems as a result of occupational exposure to several pesticides, depending on exposure duration, frequency, PPE use, and hygienic behavior. The outcomes of >1 and >2 neurotic symptoms, which may have poor clinical relevance, and mental disorder presented consistently for the majority of pesticides. All functional pesticide groups under our investigation showed a positive association with neurotic symptoms. The correlation between pesticide families may explain these findings. In our study, the history of overall herbicide exposure at high levels was related to mental disorder. Such association was also found in chloroacetamide/anilide herbicides, glyphosate, paraquat, and 2,4-D. Similarly, previous studies revealed a strong association between herbicide use and diagnosed depression [14,16]. The chemical paraquat dichloride was discovered to be a significant predictor of depressive symptoms [11]. Mental ill-health symptom scores were related to exposure to phenoxy herbicides compounds [26]. Moreover, for multiple pesticide analysis, it revealed an association between glyphosate exposure and having at least two neurotic symptoms. This raises the possibility that long-term and high-intensity glyphosate exposure may have a negative impact on farmers’ mental health.

In terms of insecticide exposure, the history of five organochlorine exposure was associated with mental disorder. This is consistent with the previous research revealing a link between organochlorine insecticides, particularly dieldrin, and depression [13,14]. In a model with multiple pesticides, a strong association between endosulfan exposure and mental disorder suggested that this chemical may have an effect on mental health. Similarly, history of exposure to nine organophosphates and methyl parathion insecticides were linked to increased disorders. These agricultural pesticides can cause neuropsychiatric disorders such as depression and anxiety due to a significant decrease in red blood cell cholinesterase activity [7]. Our findings are consistent with those of previous studies that organophosphate insecticides such as parathion were associated with anxiety and depression [10,13,14]. In the single pesticide model, farmers who used chlorpyrifos were at a higher risk of neurotic symptoms than those who did not use them. This supported a prospective study involving 55,071 pesticide applicators in Iowa and North Carolina, which suspects a link between chlorpyrifos and depression [27].

Use of carbamate insecticide was related to an increased risk of mental disorder. Similar to organophosphates, carbamates demonstrate the same of mode of action. This could have similar direct effects. This is consistent with the findings of Beseler et al. [14], who showed a strong association between ever having used carbamates and diagnosed depression in male farmer applicators. According to our findings, methomyl, carbofuran, and carbaryl are suspected to be linked to mental health problems. The current study found a marginal association between mental disorder and exposure to permethrin/cypermethrin (AOR = 2.60) and imidacloprid (AOR = 2.65). This is consistent with the findings of Campos et al. [9], who discovered a higher risk of pyrethroid exposure among rural population reported depression (OR = 1.80). Our study supported a case study report indicating that imidacloprid poisoning, which acts on the nervous system of insects and mammals via nicotinic acetylcholine receptors, can cause depression [28]. High-level exposure to abamectin/emamectin benzoate was marginally associated with approximately two times higher odds of probable mental disorder (AOR = 2.20). This may be supported by a study that was the first to report memory deficit and depressive behavior in mammal experimental models after chronic abamectin exposure, assuming the interaction with GABA receptors [29]. A previous study also showed evidence of an association between major depressive disorders and GABAergic deficits [30]. However, avermectin exposure in this study is likely to be lower than neonicotinoid exposure in terms of lifetime application days. Our research suggested that neonicotinoids and avermectins might also pose a risk.

For fungicides, mental disorder was related to overall exposure, dithiocarbamates, and bordeaux mixture/CuSO_4_. Our findings found a marginal association between metalaxyl exposure and probable mental disorder (AOR = 2.69). This is supported by a previous study, which discovered an association between ever having used fungicides and a physician-diagnosed depression in farmer applicators [14]. According to our findings, high-level exposure to dithiocarbamates, bordeaux mixture/CuSO_4_, and metalaxyl might be harmful to mental health.

The farmers who were exposed to any rodenticides and molluscicides are more likely to have mental disorder. Although these explanations are limited, some documents have reported their toxicity, which may be relevant to mental health issues. The chemical compounds in these two pesticide classes may be highly toxic. Zinc phosphide (WHO class Ib: highly hazardous) is the most commonly used rodenticide imported into Thailand, whereas the molluscicides were often clonitralide (WHO class U: unlikely to present acute hazard in normal use) and metaldehyde (WHO class II: moderately hazardous). Early signs of exposure to zinc phosphide include anorexia and depression [31]. A study showed evidence of neuropsychiatric signs and symptoms from exposure to zinc phosphide pesticide in workers [32]. Initial clinical signs of exposure to metaldehyde may include anxiety [33]. High acute exposure can also lead to depression and drowsiness, whereas long-term exposure may result in dermatitis and affect brain function in humans [34]. Furthermore, carbamate pesticides such as aldicarb, carbaryl, and methomyl are used as molluscicides, resulting in cholinesterase inhibition. In the past, Thai farmers also utilized endosulfan insecticide to get rid of channeled apple snails. Our findings supported this information, demonstrating a strong correlation between molluscicide lifetime application days and endosulfan. However, farmers’ use of a diverse range of chemicals over their lifetime makes it difficult to identify specific agents associated with disease [5]. The effects of prolonged exposure on mental health may need to be documented, and more evidence is required to support our findings. 

When only pesticide users were considered, self-reporting of previous pesticide poisoning was strongly associated with a greater risk of mental disorder. This is similar to previous studies, which found that a history of pesticide poisoning was positively associated with depression and anxiety [8,9,10,11,12] and suicidal ideation [35]. Moreover, depression was linked to the severity of poisoning symptoms [11]. These findings suggested that farmers, particularly those who have been poisoned by pesticides, should be monitored under health surveillance to track their mental health.

The most common symptoms such as poor sleep and headache indicated prominent somatic symptoms in Thai farmers. Unlike inhabitants of Western countries, those of Asian descent tend to show these symptoms because depression reflects illnesses both physically and mentally [2,24]. A previous study supported our findings by revealing an association between cumulative exposure to pesticides and sleep problems, such as short sleep duration and having difficulty sleeping, among greenhouse vegetable farmers [36]. Furthermore, the study of Baumert et al. [37] showed a positive association between exposure to carbofuran and sleep apnea in male pesticide applicators. Chronic and serious headache can have significant impact on an individual’s quality of life [38]. 

Due to the low prevalence of probable mental disorder among farmers, people with neurotic symptoms or mental distress had the option to refuse to participate in the study, resulting in lower/underestimation of reporting. Despite the fact that SRQ-20 has been used in some studies in Thailand [39,40], there is no cut-off score for the Thai population. As a result, the questionnaire should be validated and tested the psychometric properties in Thai people. Another option, the use of a specific questionnaire for screening depression such as the Patient Health Questionnaire (PHQ) and anxiety such as the Generalized Anxiety Disorder (GAD), which was validated among Thai people, could be appropriate. Because of self-reports of subjective symptoms, clinical reappraisals or mental disorder diagnosis by a health professional physician may be considered.

### 4.2. Study Limitations

This study exhibits some limitations. The history of pesticide exposure could result in reporting bias and misclassification, particularly for some chemical compounds that have been banned in Thailand for a long time. Our study attempted to reduce bias by asking about trade names and illustrating pesticide pictures during the interview for easier recall. In addition, repeat interviews from a sample of participants in the Agricultural Health Study revealed high reliability for ever-/never-use of specific pesticides, and lower for duration and frequency of pesticide use [20]. Considering the low prevalence of self-reported mental disorder, the statistical power of exploratory analyses was limited, implying the possibility of false positive findings. The effects of numerous active ingredients could not be assessed because of the low reported use. Exposure to other pesticides and information on current use are not included in this study. In order to address causality, more robust designs could be required. Despite the fact that cluster sampling was used in this study, public health officers and health volunteers in some areas were unable to collect data from subjects due to time constraints and the COVID-19 pandemic. This could have an impact on the representativeness and generalizability of the farmer population. In addition, the COVID-19 pandemic may have contributed to the farmers’ anxiety and depression. Self-reported questionnaires are likely to be less reliable for people with poor mental health. However, this study provides evidence of a potential risk of mental health effects from agricultural pesticide exposure. These are concerning because of their high risk and the fact that they are still widely used currently in Thailand. It is critical for the government to review policy and pesticide legislation as well as provide pesticide guidelines for the informal sector, with a focus on promoting and improving farmers’ health and well-being. From a researcher’s view, while risk cannot be eliminated, it can be minimized. Effective strategies for preventing neurotic symptoms and treatments for mental disorder among farmers should be considered.

## 5. Conclusions

Our findings emphasized that the use of various pesticides is associated with a greater risk of mental disorder in rice, vegetable, fruit, and flower farmers. Common somatic symptoms such as insomnia, headaches, and loss of appetite were observed. These chronic symptoms can endanger the farmers’ health and have an influence on the quality of life, recognizing the significance of a pesticide-related mental health issue. As a consequence, an intervention to provide knowledge and training on the potential risks of occupational pesticide exposure as well as prevention of mental disorders among Thai farmers should be considered. Furthermore, the effects of pesticide exposure on mental health conditions should be of concern to governments.

## Figures and Tables

**Table 1:** General information of Thai farmers (n = 6974).

Socio-Demographic Factors	n (%)	Work-Related Factors	n (%)
Sex		Work experience (years) [Mean ± SD]	23.3 ± 13.0
Male	3752 (53.8%)	Rice farm	3624 (52.0%)
Female	3222 (46.2%)	Vegetable farm	2047 (29.4%)
Age (years) [Mean ± SD]	55.2 ± 11.7	Fruit farm	4508 (64.6%)
≤40 years	768 (11.0%)	Flower farm	495 (7.1%)
41–50 years	1222 (17.5%)	Agriculture land	
51–60 years	2257 (32.4%)	≤8000 m^2^	3753 (53.8%)
>60 years	2727 (39.1%)	8001–16,000 m^2^	1843 (26.4%)
Marital status		>16,000 m^2^	1378 (19.8%)
Married	5314 (76.2%)	Entry into farmland	
Single/Divorced	1660 (23.8%)	<1 time/month	498 (7.1%)
Education level		Every month	703 (10.1%)
No	984 (14.1%)	Every week	3490 (50.1%)
Primary school	4603 (66.0%)	Everyday	2283 (32.7%)
Secondary school or higher	1387 (19.9%)	History of pesticide use in agriculture	
Monthly family income		Never	929 (13.3%)
≤5000 Baht	3216 (46.1%)	Ever used	6045 (86.7%)
5001–10,000 Baht	2770 (39.7%)	Mixer (n = 6045)	2715 (44.9%)
>10,000 Baht	988 (14.2%)	Sprayer (n = 6045)	4540 (75.1%)
Currently smoking	1273 (18.3%)	PPE score (n = 6045) [Mean ± SD]	0.33 ± 0.20
Currently drinking alcohol	2233 (32.0%)	Bathing after application (n = 6045)	
Distance from home to nearest farm		Always	5557 (91.9%)
<100 m	928 (13.3%)	Sometimes	353 (5.9%)
100–300 m	1174 (16.8%)	Never	135 (2.2%)
300 m–1 km	2353 (33.8%)	Pesticide poisoning (n = 6045)	
2–5 km	1778 (25.5%)	No	5000 (82.7%)
>5 km	741 (10.6%)	Yes	1045 (17.3%)

**Table 2:** History of individual pesticide use among Thai famers (n = 6045).

	Pesticide	WHO Class	n (%)	Lifetime Application Days: Percentile (P)				
				25th	50th	75th	90th	95th
	**Pesticide**		6045 (100%)	25	73	219	510	786
**1.**	**Herbicide**		5439 (90.0%)	9	22	73	170	262
1.1	Glycine: Glyphosate	III	4489 (74.3%)	40	103	290	590	990
1.2	Bipyridylium: Paraquat	II	3953 (65.4%)	40	103	368	590	1200
1.3	Phenoxy: 2,4-D	II	1019 (16.9%)	25	100	225	590	840
1.4	Chloroacetamide/Anilide:		714 (10.2%)	51	109	396	990	1980
1.4.1	Alachlor	II	463 (7.7%)	51	109	368	767	1628
1.4.2	Butachlor/Propanil	III/II	380 (6.3%)	51	116	396	990	1628
**2.**	**Insecticide**		5519 (91.3%)	12	33	84	191	262
2.1	Organochlorine (OC):		797 (13.2%)	40	100	236	749	1110
2.1.1	Endosulfan	II	555 (9.2%)	40	100	225	457	767
2.1.2	DDT	II	246 (4.1%)	40	109	368	767	990
2.1.3	Chlordane	II	66 (1.1%)	25	128	375	789	1478
2.1.4	Heptachlor	O	43 (0.7%)	51	116	368	643	767
2.1.5	Dieldrin/Aldrin	O/O	41 (0.7%)	49	116	663	1500	1628
2.2	Organophosphate (OP):		1367 (22.6%)	40	103	290	792	1628
2.2.1	Chlorpyrifos	II	524 (8.7%)	56	116	290	679	1200
2.2.2	Methyl parathion	Ia	521 (8.6%)	40	100	290	590	840
2.2.3	Methamidophos	Ib	252 (4.2%)	51	116	236	590	893
2.2.4	Dichlorvos	Ib	202 (3.3%)	36	40	40	78	282
2.2.5	Monocrotophos	Ib	85 (1.4%)	56	173	590	661	990
2.2.6	EPN	Ia	80 (1.3%)	19	100	389	840	1628
2.2.7	Mevinphos	Ia	49 (0.8%)	25	109	302	840	1234
2.2.8	Dicrotophos	Ib	42 (0.7%)	40	110	390	767	1509
2.2.9	Profenofos	II	38 (0.6%)	51	116	590	2325	3000
2.3	Carbamate (CM):		1672 (27.7%)	40	100	282	780	1535
2.3.1	Methomyl	Ib	811 (13.4%)	40	100	236	590	990
2.3.2	Carbofuran	Ib	607 (10.0%)	25	56	116	590	930
2.3.3	Carbaryl	II	556 (9.2%)	40	78	225	457	840
2.3.4	Carbosulfan	II	389 (6.4%)	51	109	368	590	840
2.4	PY: Permethrin/Cypermethrin	II/II	318 (5.3%)	51	225	525	2100	2100
2.5	NN: Imidacloprid	II	359 (5.9%)	51	100	236	840	2100
2.6	AV: Abamectin/Emamectin	Ib	2900 (48.0%)	56	116	368	590	767
**3.**	**Fungicide**		3917 (64.8%)	17	38	94	203	313
3.1	Phenylamide: Metalaxyl	II	345 (5.7%)	56	116	396	767	990
3.2	Inorganic: Bordeaux mixture/CuSO_4_	II	246 (4.1%)	78	212	457	1053	1200
3.3	Dithiocarbamate (DT):		728 (12.0%)	51	109	236	668	990
3.3.1	Mancozeb	U	547 (9.0%)	51	100	225	457	767
3.3.2	Maneb/Zineb	U/U	161 (2.7%)	51	100	230	825	990
3.3.3	Propineb	U	151 (2.5%)	51	100	236	840	990
**4.**	**Rodenticide**		667 (11.0%)	40	100	236	590	930
**5.**	**Molluscicide**		1219 (20.2%)	40	100	173	396	840

Abbreviations: PY, pyrethroid; NN, neonicotinoid; AV, avermectin; CuSO_4_, copper sulfate. Note: Ia = Extremely hazardous; Ib = Highly hazardous; II = Moderately hazardous; III = slightly hazardous; U = Unlikely to present acute hazard in normal use; O = Obsolete as pesticide, not classified.

**Table 3:** Prevalence of neurotic symptoms and mental disorder from the Self-Reporting Questionnaire (SRQ-20) in Thai farmers.

No.	Neurotic Symptoms	All(n = 6974)	No Pesticide Group(n = 929)	Pesticide Group(n = 6045)
		n (%)	n (%)	n (%)
1.	Sleeping problems	560 (8.0%)	70 (7.5%)	490 (8.1%)
2.	Headache	427 (6.1%)	47 (5.1%)	380 (6.3%)
3.	Lack of appetite	292 (4.2%)	30 (3.2%)	262 (4.3%)
4.	Feeling nervous	242 (3.5%)	30 (3.2%)	212 (3.5%)
5.	Easily tiring	154 (2.2%)	20 (2.2%)	134 (2.2%)
6.	Poor digestion	142 (2.0%)	17 (1.8%)	125 (2.1%)
7.	Shaking hands	142 (2.0%)	13 (1.4%)	129 (2.1%)
8.	Being frightened	94 (1.3%)	11 (1.2%)	83 (1.4%)
9.	Not thinking clearly	92 (1.3%)	6 (0.6%)	86 (1.4%)
10.	Being unhappy	68 (1.0%)	12 (1.3%)	56 (0.9%)
11.	Always feeling tried	62 (0.9%)	9 (1.0%)	53 (0.9%)
12.	Work suffering	53 (0.8%)	8 (0.9%)	45 (0.7%)
13.	Difficulty with decision-making	46 (0.7%)	8 (0.9%)	38 (0.6%)
14.	Not enjoying activities	39 (0.6%)	4 (0.4%)	35 (0.6%)
15.	Stomach problems	34 (0.5%)	2 (0.2%)	32 (0.5%)
16.	Loss of interest in life	28 (0.4%)	2 (0.2%)	26 (0.4%)
17.	Crying more than normally	25 (0.4%)	3 (0.3%)	22 (0.4%)
18.	Feeling worthless	24 (0.3%)	3 (0.3%)	21 (0.3%)
19.	Thinking of ending life	24 (0.3%)	3 (0.3%)	21 (0.3%)
20.	Not feeling life is useful	21 (0.3%)	4 (0.4%)	17 (0.3%)
	Percentile 90 (≥2 symptoms)	537 (7.7%)	68 (7.3%)	469 (7.8%)
	Percentile 95 (≥3 symptoms)	303 (4.3%)	36 (3.9%)	267 (4.4%)
	Probable mental disorder	101 (1.4%)	8 (0.9%)	93 (1.5%)

**Table 4:** Adjusted odds ratios of neurotic symptoms and probable mental disorder from occupational exposure to pesticides in Thai farmers (n = 6974).

Variable	≥2 Symptoms	≥3 Symptoms	≥6 Symptoms
	AOR (95%CI)	AOR (95%CI)	AOR (95%CI)
**Pesticide poisoning (n = 6045)**			
No (n = 5000)	1	1	1
Yes (n = 1045)	8.38 (6.84, 10.27) *	8.41 (6.48, 10.92) *	7.97 (5.16, 12.31) *
**Cumulative exposure to any pesticide**			
No history of pesticide use (n = 929)	1	1	1
Low exposure (<P50) (n = 3026)	0.95 (0.71, 1.27)	0.91 (0.61, 1.34)	1.05 (0.47, 2.34)
Medium exposure (P50–P90) (n = 2424)	1.33 (0.99, 1.77)	1.31 (0.88, 1.94)	1.97 (0.90, 4.28)
High exposure (>P90) (n = 595)	1.98 (1.39, 2.82) *	3.07 (1.98, 4.75) *	6.24 (2.80, 13.89) *
**Cumulative exposure: functional group**			
**Herbicide**			
No history of pesticide use (n = 929)	1	1	1
Ever used other pesticides (n = 606)	0.89 (0.59, 1.34)	0.99 (0.58, 1.69)	1.30 (0.47, 3.62)
Low exposure (<P50) (n = 2824)	0.89 (0.66, 1.19)	0.82 (0.55, 1.23)	0.93 (0.41, 2.12)
Medium exposure (P50–P90) (n = 2078)	1.59 (1.19, 2.13) *	1.65 (1.12, 2.43) *	2.78 (1.29, 5.99) *
High exposure (>P90) (n = 537)	1.81 (1.25, 2.61) *	2.64 (1.67, 4.17) *	4.62 (2.00, 10.67) *
**Insecticide**			
No history of pesticide use (n = 929)	1	1	1
Ever used other pesticides (n = 526)	0.77 (0.49, 1.20)	0.63 (0.33, 1.19)	0.55 (0.14, 2.09)
Low exposure (<P50) (n = 2777)	0.97 (0.72, 1.30)	0.98 (0.66, 1.45)	1.58 (0.72, 3.45)
Medium exposure (P50–P90) (n = 2200)	1.51 (1.13, 2.02) *	1.58 (1.07, 2.33) *	2.40 (1.11, 5.18) *
High exposure (>P90) (n = 542)	1.61 (1.10, 2.34) *	2.23 (1.39, 3.57) *	3.28 (1.36, 7.94) *
**Fungicide**			
No history of pesticide use (n = 929)	1	1	1
Ever used other pesticides (n = 2128)	1.45 (1.08, 1.94) *	1.29 (0.87, 1.91)	1.30 (0.58, 2.92)
Low exposure (<P50) (n = 2082)	0.90 (0.65, 1.22)	1.00 (0.66, 1.52)	1.68 (0.75, 3.76)
Medium exposure (P50–P90) (n = 1449)	1.22 (0.89, 1.68)	1.48 (0.98, 2.25)	3.06 (1.39, 6.70) *
High exposure (>P90) (n = 386)	1.23 (0.79, 1.93)	1.66 (0.95, 2.91)	2.80 (1.03, 7.59) *
**Rodenticide**			
No history of pesticide use (n = 929)	1	1	1
Ever used other pesticides (n = 5378)	1.05 (0.80, 1.38)	1.01 (0.70, 1.46)	1.32 (0.6 2.79)
Low exposure (<P50) (n = 334)	1.36 (0.85, 2.16)	1.65 (0.92, 2.98)	3.56 (1.34, 9.45) *
High exposure (>P50) (n = 333)	3.95 (2.73, 5.71) *	5.99 (3.82, 9.39) *	10.89 (4.83, 24.56) *
**Molluscicide**			
No history of pesticide use (n = 929)	1	1	1
Ever used other pesticides (n = 4826)	0.94 (0.71, 1.24)	0.85 (0.58, 1.24)	0.94 (0.43, 2.03)
Low exposure (<P50) (n = 629)	1.58 (1.09, 2.28) *	1.85 (1.14, 2.99) *	4.08 (1.73, 9.63) *
High exposure (>P50) (n = 590)	3.15 (2.26, 4.40) *	4.80 (3.15, 7.31) *	9.63 (4.38, 21.18) *
**Cumulative exposure: chemical class**			
**Glycine: Glyphosate**			
No history of pesticide use (n = 929)	1	1	1
Ever used other pesticides (n = 1695)	0.89 (0.64, 1.22)	0.81 (0.52, 1.26)	1.06 (0.44, 2.53)
Low exposure (<P50) (n = 2129)	0.86 (0.63, 1.17)	0.85 (0.56, 1.30)	1.09 (0.47, 2.52)
Medium exposure (P50–P90) (n = 1782)	1.75 (1.30, 2.35) *	1.90 (1.28, 2.81) *	3.09 (1.43, 6.67) *
High exposure (>P90) (n = 439)	1.97 (1.35, 2.87) *	2.63 (1.64, 4.22) *	4.51 (1.91, 10.65) *
**Bipyridylium: Paraquat**			
No history of pesticide use (n = 929)	1	1	1
Ever used other pesticides (n = 2092)	1.04 (.77, 1.41)	0.95 (0.63, 1.44)	1.33 (0.59, 3.04)
Low exposure (<P50) (n = 1978)	0.85 (0.61, 1.16)	0.87 (0.57, 1.33)	0.97 (0.41, 2.29)
Medium exposure (P50–P90) (n = 1595)	1.80 (1.33, 2.43) *	2.03 (1.37, 3.01) *	3.35 (1.55, 7.25) *
High exposure (>P90) (n = 380)	1.58 (1.04, 2.40) *	2.14 (1.28, 3.59) *	4.15 (1.69, 10.20) *
**Phenoxy: 2,4-D**			
No history of pesticide use (n = 929)	1	1	1
Ever used other pesticides (n = 5026)	1.06 (0.81, 1.40)	1.07 (0.74, 1.54)	1.58 (0.75, 3.34)
Low exposure (<P50) (n = 527)	1.20 (0.79, 1.81)	0.97 (0.54, 1.76)	1.29 (0.41, 3.99)
High exposure (>P50) (n = 492)	2.58 (1.81, 3.67) *	3.74 (2.41, 5.82) *	5.92 (2.59, 13.51) *
**Chloroacetamide/Anilide**			
No history of pesticide use (n = 929)	1	1	1
Ever used other pesticides (n = 5331)	1.06 (0.81, 1.40)	1.10 (0.76, 1.58)	1.55 (0.73, 3.26)
Low exposure (<P50) (n = 370)	1.67 (1.09, 2.57) *	1.65 (0.93, 2.94)	2.40 (0.82, 7.05)
High exposure (>P50) (n = 344)	2.93 (2.00, 4.29) *	3.70 (2.29, 5.99) *	7.41 (3.17, 17.31) *
**Organochlorine**			
No history of pesticide use (n = 929)	1	1	1
Ever used other pesticides (n = 5248)	1.02 (0.78, 1.34)	0.99 (0.68, 1.43)	1.36 (0.64, 2.88)
Low exposure (<P50) (n = 401)	1.77 (1.17, 2.66) *	1.95 (1.14, 3.34) *	2.89 (1.06, 7.86) *
High exposure (>P50) (n = 396)	3.05 (2.12, 4.39) *	4.63 (2.95, 7.25) *	9.26 (4.08, 20.99) *
**Organophosphate**			
No history of pesticide use (n = 929)	1	1	1
Ever used other pesticides (n = 4678)	1.09 (0.83, 1.44)	1.07 (0.74, 1.55)	1.50 (0.71, 3.17)
Low exposure (<P50) (n = 695)	0.85 (0.56, 1.28)	1.07 (0.63, 1.81)	1.46 (0.54, 3.96)
High exposure (>P50) (n = 672)	2.27 (1.62, 3.18) *	2.88 (1.87, 4.42) *	5.35 (2.40, 11.95) *
**Carbamate**			
No history of pesticide use (n = 929)	1	1	1
Ever used other pesticides (n = 4373)	0.96 (0.72, 1.26)	0.97 (0.66, 1.41)	1.36 (0.64, 2.91)
Low exposure (<P50) (n = 868)	1.19 (0.83, 1.70)	1.00 (0.61, 1.66)	1.38 (0.56, 3.64)
High exposure (>P50) (n = 804)	2.60 (1.89, 3.58) *	3.37 (2.23, 5.09) *	5.97 (2.71, 13.19) *
**Pyrethroid: Permethrin/Cypermethrin**			
No history of pesticide use (n = 929)	1	1	1
Ever used other pesticides (n = 5727)	1.18 (0.90, 1.54)	1.22 (0.85, 1.75)	1.87 (0.90, 3.91)
Ever used pyrethroid (n = 318)	1.48 (0.93, 2.36)	2.11 (1.20, 3.69) *	2.60 (0.89, 7.63)
**Neonicotinoid: Imidacloprid**			
No history of pesticide use (n = 929)	1	1	1
Ever used other pesticides (n = 5686)	1.16 (0.88, 1.52)	1.23 (0.86, 1.77)	1.87 (0.90, 3.91)
Ever used imidacloprid (n = 359)	1.79 (1.16, 2.75) *	1.72 (0.96, 3.10)	2.65 (0.90, 7.80)
**Avermectin: Abamectin/Emamectin**			
No history of pesticide use (n = 929)	1	1	1
Ever used other pesticides (n = 3145)	1.23 (0.93, 1.63)	1.31 (0.90, 1.91)	2.05 (0.96, 4.35)
Low exposure (<P50) (n = 1521)	0.79 (0.56, 1.11)	0.82 (0.52, 1.29)	1.29 (0.54, 3.12)
High exposure (>P50) (n = 1379)	1.57 (1.15, 2.15) *	1.64 (1.09, 2.48) *	2.20 (0.97, 4.99)
**Dithiocarbamate**			
No history of pesticide use (n = 929)	1	1	1
Ever used other pesticides (n = 5317)	1.16 (0.88, 1.52)	1.21 (0.84, 1.74)	1.78 (0.85, 3.73)
Low exposure (<P50) (n = 364)	0.81 (0.48, 1.37)	1.05 (0.55, 2.02)	1.78 (0.57, 5.55)
High exposure (>P50) (n = 364)	2.05 (1.36, 3.07) *	2.24 (1.32, 3.82) *	4.14 (1.60, 10.68) *
**Phenylamide: Metalaxyl**			
No history of pesticide use (n = 929)	1	1	1
Ever used other pesticides (n = 5700)	1.15 (0.88, 1.51)	1.22 (0.85, 1.75)	1.86 (0.89, 3.89)
Ever used metalaxyl (n = 345)	1.93 (1.27, 2.94) *	1.98 (1.14, 3.45) *	2.69 (0.96, 7.56)
**Inorganic: Bordeaux mixture/CuSO_4_**			
No history of pesticide use (n = 929)	1	1	1
Ever used other pesticides (n = 5799)	1.15 (0.87, 1.50)	1.19 (0.83, 1.71)	1.75 (0.84, 3.66)
Ever used CuSO_4_ (n = 246)	2.35 (1.50, 3.68) *	2.94 (1.68, 5.15) *	5.82 (2.24, 15.10) *

The model was adjusted for sex, age, marital status, education, smoking, and drinking alcohol. * Significance at 0.05 level.

**Table 5:** Association of individual pesticide exposure with mental disorder in Thai farmers (n = 6974).

Pesticide Active Ingredient	≥2 Symptoms	≥3 Symptoms	≥6 Symptoms
(Ever Used vs. Never Used)	AOR (95%CI)	AOR (95%CI)	AOR (95%CI)
**Single model**			
Glyphosate (n = 4489)	1.57 (1.24, 2.00) *	1.78 (1.29, 2.46) *	2.11 (1.19, 3.76) *
Paraquat (n = 3953)	1.24 (1.01, 1.52) *	1.51 (1.14, 2.01) *	1.67 (1.01, 2.74) *
2,4-D (n = 1019)	1.74 (1.38, 2.18) *	2.12 (1.60, 2.81) *	2.24 (1.41, 3.54) *
Endosulfan (n = 555)	2.27 (1.74, 2.96) *	2.97 (2.15, 4.10) *	4.34 (2.66, 7.09) *
Chlorpyrifos (n = 524)	1.36 (1.00, 1.85) *	1.58 (1.08, 2.31) *	1.51 (0.79, 2.88)
Methyl parathion (n = 521)	3.13 (2.42, 4.04) *	3.67 (2.68, 5.03) *	4.44 (2.71, 7.29) *
Methomyl (n = 811)	2.98 (2.39, 3.72) *	3.22 (2.43, 4.27) *	3.45 (2.18, 5.45) *
Carbofuran (n = 607)	1.88 (1.43, 2.47) *	2.62 (1.89, 3.63) *	3.31 (1.97, 5.57) *
Carbaryl (n = 556)	1.77 (1.34, 2.35) *	1.89 (1.32, 2.71) *	1.94 (1.06, 3.54) *
Abamectin/Emamectin (n = 2900)	0.94 (0.77, 1.14)	0.92 (0.72, 1.19)	0.85 (0.56, 1.30)
Mancozeb (n = 547)	1.11 (0.80, 1.54)	1.11 (0.73, 1.71)	1.31 (0.67, 2.56)
**Multiple model**			
Glyphosate (n = 4489)	1.35 (1.03, 1.75) *	1.38 (0.96, 1.97)	1.57 (0.83, 2.96)
Paraquat (n = 3953)	0.86 (0.68, 1.09)	0.99 (0.72, 1.36)	0.99 (0.57, 1.71)
2,4-D (n = 1019)	1.21 (0.94, 1.55)	1.35 (0.98, 1.85)	1.26 (0.75, 2.11)
Endosulfan (n = 555)	1.27 (0.93, 1.74)	1.52 (1.04, 2.23) *	2.27 (1.26, 4.08) *
Chlorpyrifos (n = 524)	0.96 (0.68, 1.35)	1.07 (0.70, 1.64)	0.98 (0.47, 2.03)
Methyl parathion (n = 521)	2.05 (1.53, 2.76) *	2.04 (1.41, 2.95) *	2.26 (1.26, 4.06) *
Methomyl (n = 811)	2.22 (1.71, 2.89) *	2.00 (1.42, 2.81) *	1.74 (0.98, 3.09)
Carbofuran (n = 607)	1.11 (0.82, 1.50)	1.46 (1.01, 2.11) *	1.66 (0.93, 2.99)
Carbaryl (n = 556)	1.14 (0.83, 1.57)	1.07 (0.71, 1.60)	1.02 (0.52, 2.02)
Abamectin/Emamectin (n = 2900)	0.84 (0.69, 1.03)	0.79 (0.61, 1.03)	0.67 (0.43, 1.06)
Mancozeb (n = 547)	0.74 (0.52, 1.05)	0.66 (0.42, 1.05)	0.77 (0.38, 1.59)

The model was adjusted for history of pesticide use in agriculture, sex, age, marital status, education, smoking, and drinking alcohol. * Significance at 0.05 level.

## Data Availability

Not applicable.
